# Systematic Production of Inactivating and Non-Inactivating Suppressor Mutations at the *relA* Locus That Compensate the Detrimental Effects of Complete *spoT* Loss and Affect Glycogen Content in *Escherichia coli*

**DOI:** 10.1371/journal.pone.0106938

**Published:** 2014-09-04

**Authors:** Manuel Montero, Mehdi Rahimpour, Alejandro M. Viale, Goizeder Almagro, Gustavo Eydallin, Ángel Sevilla, Manuel Cánovas, Cristina Bernal, Ana Belén Lozano, Francisco José Muñoz, Edurne Baroja-Fernández, Abdellatif Bahaji, Hirotada Mori, Francisco M. Codoñer, Javier Pozueta-Romero

**Affiliations:** 1 Instituto de Agrobiotecnología, UPNA/CSIC/Gobierno de Navarra, Mutiloabeti, Nafarroa, Spain; 2 Instituto de Biología Molecular y Celular de Rosario (IBR, CONICET), Departamento de Microbiología, Facultad de Ciencias Bioquímicas y Farmacéuticas, Universidad Nacional de Rosario, Rosario, Argentina; 3 Departamento de Bioquímica y Biología Molecular e Inmunología, Facultad de Química, Campus de Excelencia Internacional Regional “Campus Mare Nostrum”, Universidad de Murcia, Murcia, Spain; 4 Graduate School of Biological Sciences, Nara Institute of Science and Technology, Nara, Japan; 5 Lifesequencing S.L., Paterna, Valencia, Spain; University of Rochester Medical Center, United States of America

## Abstract

In *Escherichia coli,* ppGpp is a major determinant of growth and glycogen accumulation. Levels of this signaling nucleotide are controlled by the balanced activities of the ppGpp RelA synthetase and the dual-function hydrolase/synthetase SpoT. Here we report the construction of *spoT* null (Δ*spoT*) mutants obtained by transducing a Δ*spoT* allele from Δ*relA*Δ*spoT* double mutants into *relA^+^* cells. Iodine staining of randomly selected transductants cultured on a rich complex medium revealed differences in glycogen content among them. Sequence and biochemical analyses of 8 Δ*spoT* clones displaying glycogen-deficient phenotypes revealed different inactivating mutations in *relA* and no detectable ppGpp when cells were cultured on a rich complex medium. Remarkably, although the co-existence of Δ*spoT* with *relA* proficient alleles has generally been considered synthetically lethal, we found that 11 Δ*spoT* clones displaying high glycogen phenotypes possessed *relA* mutant alleles with non-inactivating mutations that encoded stable RelA proteins and ppGpp contents reaching 45–85% of those of wild type cells. None of the Δ*spoT* clones, however, could grow on M9-glucose minimal medium. Both Sanger sequencing of specific genes and high-throughput genome sequencing of the Δ*spoT* clones revealed that suppressor mutations were restricted to the *relA* locus. The overall results (a) defined in around 4 nmoles ppGpp/g dry weight the threshold cellular levels that suffice to trigger net glycogen accumulation, (b) showed that mutations in *relA*, but not necessarily inactivating mutations, can be selected to compensate total SpoT function(s) loss, and (c) provided useful tools for studies of the *in* *vivo* regulation of *E. coli* RelA ppGpp synthetase.

## Introduction

In conditions in which carbon sources are in excess and other nutrients are deficient, *Escherichia coli* and many bacterial species accumulate glycogen, a branched homopolysaccharide of α-1,4-linked glucose subunits with α-1,6-linkages at the branching points [1], [2]. Synthesized by glycogen synthase (GlgA) using ADP-glucose as the glucosyl moiety donor, the exact role of glycogen in bacterial physiology is still not well defined, but several works have linked this polysaccharide to a reserve source of energy and carbon, environmental survival, intestine colonization, virulence, etc. [1]–[3].

Glycogen metabolism in *E. coli* is regulated by a complex and still not well defined assemblage of transcriptional and post-transcriptional factors that are adjusted to the nutritional status of the cell [1], [2]. We have recently re-evaluated the genetic basis of glycogen gene expression and regulation [2], [4]–[8] and found that, contrary to the long prevailing view of the existence of two separate and differentially regulated *glgBX* (encompassing the genes coding for glycogen branching (GlgB) and debranching (GlgX) enzymes), and *glgCAP* (encoding the GlgC and GlgA anabolic enzymes as well as the catabolic glycogen phosphorylase (GlgP)) glycogen operons [1], *E. coli* glycogen genes are organized in a single *glgBXCAP* operon [7]. In a search for main intracellular signals regulating glycogen metabolism in this model organism, we found that *glgBXCAP* glycogen gene expression and consequent glycogen accumulation were highly impaired in cells bearing a *relA* null ((Δ*relA*) allele [7]. These results reinforced long proposals that the RelA product ppGpp controls glycogen biosynthesis in *E. coli* [1], [2]. Moreover, our studies indicated that *E. coli* glycogen content was also reduced by mutations in other regulatory genes with known links to ppGpp such as *crp*, *rpoS*, *glgS*, *rpoN*, *phoPQ*, *fis*, *dksA*, *hfq*, etc. [4], [5]. Since ppGpp lies at the top of the network governing global gene expression in response to nutrient availability [9]–[12], the overall observations pointed to this nucleotide as a master controller of *E. coli* glycogen metabolism.

Accumulation of ppGpp constitutes the hallmark of the so-called stringent response, which is elicited in *E. coli* and many other organisms facing amino acid starvation [9]–[12]. This pleiotropic response was originally characterized by the rapid (“stringent”) inhibition of stable (rRNA and tRNA) synthesis, downregulation of the translational apparatus, and growth arrest [9]–[12]. That ppGpp was the main controller of this response was supported by the inverse correlation generally found between ppGpp basal levels and bacterial growth rates [9]–[14]. Years of experimentation indicate now that ppGpp actions extend to fundamental aspects of bacterial biology including adaptability, survival, stress responses, biofilm production, evolvability, pathogenesis, etc. [9]–[12].

*E. coli* ppGpp cellular levels are regulated by the balanced activities of RelA and SpoT, which share around 31% of amino acid sequence identity and can both catalyze the phosphorylation of GDP (or GTP) to ppGpp (or pppGpp) using ATP as the phosphoryl donor [9]–[12]. RelA represents the main source of ppGpp upon amino acid starvation, a condition in which lack of aminoacyl-tRNAs activates ribosome-bound RelA to synthesize this nucleotide [9]–[12]. SpoT, on the contrary, can produce ppGpp under deprivation of sources other than amino acids such as energy and carbon, phosphate, iron, and fatty acids [9]–[13]. Most importantly, SpoT is also endowed with ppGpp hydrolase activity, providing an essential function for the regulation of basal ppGpp levels in the cell [9]–[14]. The dual activity of SpoT makes it especially suitable for this purpose, since a small balance shift between synthesis and hydrolysis would exert an immediate impact on cellular ppGpp accumulation [9]–[12]. ppGpp binds directly to the RNA polymerase, although it can act synergistically or antagonistically with DksA (or other auxiliary factors) to exert regulatory actions [9]–[12]. This binding alters RNA polymerase performance resulting in decreased transcription at stringent promoters [9]–[12]. Moreover, increased transcription at a few RpoD- and alternative sigma factor (RpoN, RpoS, RpoF, and RpoE)-dependent promoters also occur, resulting in the activation of a number of biosynthetic operons as well as stress/survival functions [9]–[12].

Besides the strident effects provoked by the rapidly elevated levels of ppGpp resulting upon amino acid starvation, less pronounced modifications in ppGpp basal levels (resulting from subtle regulations of the balance between RelA and SpoT activities provoked by limitation of certain nutrients) are thought to have important physiological roles during cell growth [9]–[18]. Prevalent ideas are that, as the nutritional quality of the medium deteriorates and growth rate consequently diminishes, cells may be continuously adjusting ppGpp basal levels to properly allocate resources between biosynthetic and stress/survival functions [9]–[18]. In this context, recent experimental evidence has shown the existence of ppGpp-mediated hierarchical responses in global transcription patterns to amino acid starvation in *E. coli* [16]–[18]. In one of these works [17] two main regulatory circuits induced at different ppGpp cellular concentrations provoked by isoleucine limitation were identified. Thus, low basal ppGpp levels caused during isoleucine shortage induced the Lrp regulon and amino acid biosynthetic operons necessary for maintaining active growth, while much higher ppGpp levels resulting from isoleucine depletion induced growth arrest, and fully activated the RpoS-mediated general stress response [17], [18].

Different procedures are generally used to investigate the regulatory involvement of ppGpp in a given process, all having both advantages and drawbacks [9]. One common procedure involves the starvation of a *relA* ^+^ strain for a given amino acid. This results in rapid and large increases in cellular ppGpp levels with concomitant and measurable consequences, but has the disadvantage of prematurely arresting cell growth and of inducing a variety of undesirable stress responses [9], [16], [17]. Still, the combination of this procedure with the use of isogenic bacteria bearing highly defective or null *relA* alleles (i.e., “relaxed” strains), either alone or in combination with deficient *spoT* alleles, represents another commonly used strategy to uncover ppGpp roles in a given process [9]. Single *relA* mutants can still produce ppGpp due to SpoT activity, but the simultaneous deletion of *relA* and *spoT* genes (Δ*relA*Δ*spoT* double mutants) generates bacteria totally devoid of ppGpp (ppGpp^0^ cells) [9]–[12], [19], [20]. The latter mutants exhibit auxotrophies for multiple amino acids, apparently because ppGpp is required for the effective transcription of the corresponding biosynthetic genes [9]–[12], [19], [20]. Another genetic approach to manipulate ppGpp basal levels includes the use of defective *spoT* alleles [9], [14], [15], [20]. Still, severe *spoT* mutants were generally assumed to survive only in highly deficient *relA* backgrounds since conditional lethality was observed when the corresponding *spoT* alleles were introduced into *relA* ^+^ cells, an effect attributed to the resulting high ppGpp cellular levels promoted by unchecked RelA activity [9], [14], [20]. And last but not least, different studies have indicated that the richer the culture medium, the lower the cellular ppGpp levels [9], [13], [14]. It follows that the use of enriched culture media could provide a useful means for maintaining low ppGpp basal levels during cell growth. Moreover, the use of enriched media could also help differentiating changes elicited by small increases on basal ppGpp levels (which are expected as the nutritional quality of the medium slowly deteriorates) from changes derived from abrupt and large ppGpp increases that generally accompany the rapid depletion of a particular essential nutrient [9].

Considering the role of ppGpp as a main regulator of *E. coli* glycogen metabolism and the function of this polysaccharide as a reserve source of energy and carbon for long-term survival discussed above, we found of interest to define with precision the threshold ppGpp levels that promote net glycogen accumulation. Towards this end we carried out sensitive HPLC/MS-based analyses of ppGpp contents and measured glycogen gene expression and glycogen accumulation in *E. coli* BW25113 cells growing in a glucose-containing rich complex medium known as Kornberg medium (KM) [21], [22]. KM was originally utilized to grow *E. coli* cells for the isolation of DNA polymerase [21], and has since provided a valuable tool for bacterial glycogen metabolism research [1], [2], [4]–[8], [22]. Moreover, in the course of our studies we found that a Δ*spoT::Spc* null allele could be transduced into BW25113 *relA^+^* cells, and that the different *spoT*-less clones selected under the employed conditions could be separated in two main categories: i) Δ*spoT* clones with low glycogen content due to reduced glycogen gene expression, no detectable ppGpp, and *relA* alleles bearing different inactivating mutations; ii) Δ*spoT* clones with high glycogen content due to increased glycogen gene expression, significant ppGpp accumulation, and different *relA* alleles bearing non-inactivating mutations. Notably, none of these mutants could grow in M9-glucose, indicating the essentiality of SpoT function(s) for this growth mode. Also, high-throughput genome sequencing of 5 Δ*spoT* clones supported the idea that suppressor mutations of SpoT function(s) loss were centered at the *relA* locus. The overall results (a) served to define the threshold levels required to trigger net glycogen accumulation in *E. coli* to approximately 4 nmoles ppGpp/g dry weight (DW) (b) showed that not necessarily inactivating mutations in *relA* can be selected to compensate the total loss of SpoT function(s), (c) pointed to the possible occurrence of mechanism(s), other than SpoT-mediated ppGpp breakdown, that prevent accumulation of toxic ppGpp levels, and (d) provide an useful method for the systematic production of *relA* mutant alleles encoding mutant variants with significant ppGpp synthesizing capabilities. These mutants could serve useful purposes for the study of many basic questions related to RelA structure/function regulation and ppGpp biological roles. To our knowledge this is the first work describing a procedure for the systematic production of *spoT* null mutants of *E. coli* containing *relA* suppressor alleles encoding active RelA proteins.

## Materials and Methods

### *E. coli* strains and culture conditions

Strains used in this work are shown in **Table 1**. The *E. coli* K-12 BW25113 strain (*lacI* ^q^ *rrnB*_T14_ Δ*lacZ*_WJ16_ *hsdR514* Δ*araBAD*_AH33_ Δ*rhaBAD*_LD78_) and its isogenic Δ*relA* mutants were obtained from the Keio collection [23]. To analyze glycogen gene expression, glycogen accumulation, and ppGpp levels, cells were grown at 37°C with rapid gyratory shaking in liquid KM medium (1.1% K_2_HPO_4_, 0.85% KH_2_PO_4_, 0.6% Difco yeast extract from Difco, 50 mM glucose) supplemented with the appropriate selection antibiotics after inoculation with 1 volume of an overnight culture per 100 volumes of fresh culture medium. Bacterial growth was followed spectrophotometrically by measuring the Abs_600_ of the cultures at the different growth times indicated in the Figures. Growth in minimal medium was tested as described below using liquid M9 medium (95 mM Na_2_HPO_4_/44 mM KH_2_PO_4_/17 mM NaCl/37 mM NH_4_Cl/0.1 mM CaCl_2_/2 mM MgSO_4_) supplemented with 50 mM glucose. Solid KM was prepared by addition of 1.5% bacteriological agar to liquid KM before autoclaving.

**Table 1:** *E. coli* strains used in this study.

Designation	Description	Source	Comments
WT	BW25113 (*lacI* ^q^ *rrnB*_T14_Δ*lacZ*_WJ16_ *hsdR514* Δ*araBAD*_AH33_ Δ*rhaBAD*_LD78_)	Keio collection [23]	-
Δ*relA*	BW25113 Δ*relA::*Km^R^	Keio collection [23]	*rpoS, dksA, rsd, ssrS, rpoC, rpoZ* and *rpoB* sequences are identical to the corresponding genes in BW25113 (as determined by Sanger sequencing)
ΔrelA*	BW25113 Δ*relA::*Km^R^derivative in which the Km-resistance cassette was removed (Km^S^)	Montero *et al*. [7]	*rpoS, dksA, rsd, ssrS, rpoC, rpoZ* and *rpoB* sequences are identical to the corresponding genes in BW25113(as determined by Sanger sequencing)
Δ*relA*Δ*spoT*	BW25113 ΔrelA*Δ*spoT*:: Spc^R^	This work	*rpoS, dksA, rsd, ssrS, rpoC, rpoZ, rpoB, yejB* and *glnB* sequences are identical to thecorresponding genes in BW25113 (as determined by Sanger sequencing)
Δ*spoT-1*	BW25113 Δ*spoT::* Spc^R^,clone 1	This work	*- rpoS, dksA, rsd, ssrS, rpoB, rpoC, rpoZ, yejB* and *glnB* sequences are identical to the corresponding genes in BW25113 (as determined by Sanger sequencing). *relA* contains a deletion of 4 nt between positions +138 to +142 resulting in a frameshift that introduced a TAA stop codon at nt positions 202–204. Encodes a truncated polypeptide of 67 amino acids, the first 46 from the RelA N-terminal region
Δ*spoT-2*	BW25113 Δ*spoT::* Spc^R^, clone 2	This work	*- rpoS, dksA, rsd, ssrS, rpoB, rpoC, rpoZ, yejB* and *glnB* sequences are identical to the correspondinggenes in BW25113 (as determined by Sanger sequencing). *relA* contains a deletion of 2 nt at positions +360 and +361 resulting in a frameshift that introduced a TGA stop codon at nt positions 370–372. Encodes a truncated polypeptide of 123 amino acids, the first 119 from the RelA N-terminal region
Δ*spoT-3*	BW25113 Δ*spoT::* Spc^R^, clone 3	This work	*- rpoS, dksA, rsd, ssrS, rpoB, rpoC, rpoZ, yejB* and *glnB* sequences are identical to the corresponding genes in BW25113 (as determined by Sanger sequencing). *relA* contains an insertion of 1 nt at position +544 resulting in a frameshift that introduced a TAA stop codon at nt positions 559–561. Encodes a truncated polypeptide of 186 amino acids, the first 181 from the RelA N-terminal region
Δ*spoT-4*	BW25113 Δ*spoT::* Spc^R^, clone 4	This work	*- rpoS, dksA, rsd, ssrS, rpoB, rpoC, rpoZ, yejB* and *glnB* sequences are identical to the corresponding genes in BW25113 (as determined by Sanger sequencing). *relA* contains a G for T mutation at nt position +568 that introduced a premature TGA stop codon. Encodes a RelA N-terminal truncated polypeptide of 189 amino acids.
Δ***spoT-5***	BW25113 Δ*spoT::* Spc^R^, clone 5	This work	*- rpoS, dksA, rsd, ssrS, rpoB, rpoC, rpoZ, rplK, rpoD, yejB* and *glnB* sequences are identical to the corresponding genes in BW25113 (as determined by Sanger and high throughput sequencing). *relA* contains a G for T mutation at nt position +658 that introduced a premature TAA stop codon. Encodes a RelA N-terminal truncated polypeptide of 219 amino acids
Δ*spoT-6*	BW25113 Δ*spoT::* Spc^R^, clone 6	This work	*- rpoS, dksA, rsd, ssrS, rpoB, rpoC, rpoZ, yejB* and *glnB* sequences are identical to the corresponding genes in BW25113 (as determined by Sanger sequencing). *relA* contains a deletion of 7 nt at positions +1005 to +1011 resulting in a frameshift that introduced a TGA stop codon at positions 1132–1134. Encodes a truncated polypeptide of 377 amino acids, the first 335 from the RelA N-terminal region
Δ*spoT-7*	BW25113 Δ*spoT::* Spc^R^, clone 7	This work	*- rpoS, dksA, rsd, ssrS, rpoB, rpoC, rpoZ, yejB* and *glnB* sequences are identical to the corresponding genes in BW25113 (as determined by Sanger sequencing). *relA* disrupted by an insertion sequence (IS1, 777 bp) located at nt +24
Δ*spoT-8*	BW25113 Δ*spoT::* Spc^R^, clone 8	This work	*- rpoS, dksA, rsd, ssrS, rpoB, rpoC, rpoZ, yejB* and *glnB* sequences are identical to the corresponding genes in BW25113 (as determined by Sanger sequencing). *relA* allele, contains a C for A mutation at nt +774 resulting in S258R replacement, polypeptide detected by western blot
Δ*spoT-9*	BW25113 Δ*spoT::* Spc^R^, clone 9	This work	*- rpoS, dksA, rsd, ssrS, rpoB, rpoC, rpoZ, yejB* and *glnB* sequences are identical to the corresponding genes in BW25113 (as determined by Sanger sequencing). *relA* allele, contains a G for A mutation at nt +658 resulting in E220K replacement, polypeptide detected by western blot
Δ***spoT-10***	BW25113 Δ*spoT::* Spc^R^, clone 10	This work	*- rpoS, dksA, rsd, ssrS, rpoB, rpoC, rpoZ, rplK, rpoD* and *glnB* sequences are identical to the corresponding genes in BW25113 (as determined by Sanger and high throughput sequencing). *relA* allele, contains a G for A mutation at nt +1042 resulting in E348K replacement, polypeptide detected by western blot. *yejB* allele contains a G for T mutation at nt position 142 resulting in the introduction of a premature TGA stop codon, codes for a putative truncated polypeptide of 47 amino acids
Δ***spoT-11***	BW25113 Δ*spoT::* Spc^R^, clone 11	This work	*- rpoS, dksA, rsd, ssrS, rpoB, rpoC, rpoZ, rplK, rpoD, yejB* and *glnB* sequences are identical to the corresponding genes in BW25113 (as determined by Sanger and high throughput sequencing). *relA* allele, contains a A for C mutation at nt +346 resulting in I116L replacement, polypeptide detected by western blot
Δ***spoT-12***	BW25113 Δ*spoT::* Spc^R^, clone 12	This work	*- rpoS, dksA, rsd, ssrS, rpoB, rpoC, rpoZ, rplK, rpoD, yejB* and *glnB* sequences are identical to the corresponding genes in BW25113 (as determined by Sanger and high throughput sequencing). *relA* allele, contains a C for T mutation at nt +655 resulting in H219Y replacement, polypeptide detected by western blot
Δ***spoT-13***	BW25113 Δ*spoT::* Spc^R^, clone 13	This work	*- rpoS, dksA, rsd, ssrS, rpoB, rpoC, rpoZ, rplK, rpoD* and *yejB* sequences are identical to the corresponding genes in BW25113 (as determined by Sanger and high throughput sequencing). *relA* allele, contains a G for A mutation at nt +589 resulting in E197K replacement, polypeptide detected by western blot. *glnB* allele contains a 3 bp deletion eliminating nucleotides 82 to 84 resulting in GlnB lacking M28
Δ*spoT-14*	BW25113 Δ*spoT::* Spc^R^, clone 14	This work	*- rpoS, dksA, rsd, ssrS, rpoB, rpoC, rpoZ, yejB* and *glnB* sequences are identical to the corresponding genes in BW25113 (as determined by anger sequencing). *relA* allele, contains a G for C mutation at nt +287 resulting in R96P replacement, polypeptide detected by western blot
Δ*spoT-15*	BW25113 Δ*spoT::* Spc^R^, clone 15	This work	*- rpoS, dksA, rsd, ssrS, rpoB, rpoC, rpoZ, yejB* and *glnB* sequences are identical to the corresponding genes in BW25113 (as determined by Sanger sequencing). *relA* allele, contains a A for T mutation at nt +392 resulting in Q131L replacement, polypeptide detected by western blot
Δ*spoT-16*	BW25113 Δ*spoT::* Spc^R^, clone 16	This work	*- rpoS, dksA, rsd, ssrS, rpoB, rpoC, rpoZ, yejB* and *glnB* sequences are identical to the corresponding genes in BW25113 (as determined by Sanger sequencing). *relA* allele, contains a T for A mutation at nt +682 resulting in Y228N replacement, polypeptide detected by western blot
Δ*spoT-17*	BW25113 Δ*spoT::* Spc^R^, clone 17	This work	*- rpoS, dksA, rsd, ssrS, rpoB, rpoC, rpoZ, yejB* and *glnB* sequences are identical to the corresponding genes in BW25113 (as determined by Sanger sequencing). *relA* allele, contains a C for T mutation at nt +757 resulting in P253S replacement, polypeptide detected by western blot
Δ*spoT-18*	BW25113 Δ*spoT::* Spc^R^, clone 18	This work	*- rpoS, dksA, rsd, ssrS, rpoB, rpoC, rpoZ, yejB* and *glnB* sequences are identical to the corresponding genes in BW25113 (as determined by Sanger sequencing). *relA* allele, contains a G for T mutation at nt +953 resulting in G318V replacement, polypeptide detected by western blot
Δ*spoT-19*	BW25113 Δ*spoT::* Spc^R^,clone 19	This work	*- rpoS, dksA, rsd, ssrS, rpoB, rpoC, rpoZ, yejB* and *glnB* sequences are identical to the corresponding genes in BW25113 (as determined by Sanger sequencing). *RelA* allele contains a 3 bp deletion eliminating nucleotides +115 to +117 resulting in a RelA variant lacking W39, polypeptide detected by western blot
Δ*relA glgB::lacZY*	BW25113 *glgB::lacZY* transcriptional fusion inΔrelA*	Montero *et al*. [7]	-
Δ*relA*Δ*spoT glgB::lacZY*	BW25113 *glgB::lacZY* transcriptional fusion inΔ*relA*Δ*spoT*	This work	-
Δ*spoT-N glgB::lacZY*	*glgB::lacZY* transcriptionalfusion in Δ*spoT*-N (N: 1 to 19)	This work	-

Spc^R^, spectinomycin resistance. Km^R^, kanamycin resistance. Δ*spoT* clones that were subjected to high-throughput genome sequencing are indicated in **bold.**

### Generation of Δ*spoT::Spc* cells

A selectable antibiotic resistance gene was generated by PCR from a freshly isolated colony of *E. coli* MC4100 containing a Spc resistance cassette, using 60 nucleotide-long primers SPOT1 and SPOT2 (see **Table S1**) that included 40 nucleotides homology extensions for the targeted locus (*spoT*) and 20 nucleotides priming sequences for the resistance gene. This amplicon was introduced into BW25113 Δ*relA::Km* cells [23] to generate a Δ*spoT::Spc* Δ*relA::Km* double mutant following the method of Datsenko and Wanner [24]. *spoT* disruption in these cells was confirmed by PCR using the specific primer pairs (O1/O2 and O3/O4) described in **Table S2**. A double mutant strain thus obtained was used to grow P1 phage, and the lysates were used to transduce the Δ*spoT::Spc* null allele to different *E. coli* genetic backgrounds (BW25113, BL21, B/R and B) using conventional procedures. The cells were plated on solid LB medium (10 g/L tryptone, 5 g/L yeast extract, 10 g/L sodium chloride and 1.5% bacteriological agar) containing 50 µg/ml Spc, and selected clones used for further analyses were confirmed for the *spoT* disruption by PCR as above (**Table S2** and **Figure S2**). For complementation analyses, the selected Δ*spoT* clones were first transformed with the chloramphenicol resistance-conferring plasmid pCA24N-*spoT* from the ASKA collection, which directs expression of *spoT* under the control of an IPTG inducible promoter [25]. The same Δ*spoT* clones were also transformed with empty-vector constructs (pCA24N-EV) and used as controls. In all cases the transformed chloramphenicol-resistant cells were selected on LB supplemented with 25 µg/ml of this antibiotic. To test complementation by *spoT* in M9-glucose minimal medium, bacteria were first grown overnight in LB liquid medium supplemented with the corresponding antibiotics. The cells corresponding to a 1 ml aliquot of each saturated culture were then collected by centrifugation, rinsed once and resuspended in 1 ml of M9 minimal medium, and used to inoculate 100 ml of M9-glucose minimal medium kept at 37°C. The cultures were incubated under aerobic conditions at 37°C with gentle agitation, and aliquots were taken at the indicated times to measure Abs_600_ in order to follow growth kinetics.

### ppGpp extraction and HPLC/MS analysis

A novel and highly sensitive HPLC/MS ppGpp determination procedure was employed for this work. In short, ppGpp was extracted from bacterial cells grown in KM employing a two-step (quenching and extraction) procedure. In step one, bacterial cells corresponding to 20 mL of cell cultures were collected by centrifugation at the indicated culture times, treated with 20 mL of pre-cooled quenching solution (80% methanol containing 0.85% (w/vol) ammonium bicarbonate) at –50°C [26], [27], and centrifuged at –17°C for 5 min at 3000×g. The pellet thus obtained was rapidly frozen with liquid nitrogen and stored at –86°C. In step two, the pellets were resuspended in 2 mL of a nitrogen-saturated solution of acetonitrile/10 mM KH_2_ PO_4_ (pH 7.4) (3∶1; v:v) using a rotator stirrer set at 12 rpm during a 30 min period at 4°C. After centrifugation at 15500×g for 20 min at 4°C, the supernatants were extracted by vigorous agitation with 4 mL chloroform and centrifuged at 15500×g for 5 min at 4°C. This procedure was repeated three times with the upper aqueous phase, which was finally collected and filtered for subsequent analysis.

ppGpp was measured with an Agilent 1200 HPLC instrument (Agilent Technologies, California, USA) controlled by the Chemstation software, and coupled to an Agilent 6120 single quadrupole mass spectrometer with an orthogonal ESI source working in the positive mode for single ion monitoring (m/z = 604). The separation was carried out at 25°C [28] on 10 µl samples, using a ZIC-HILIC column (150×4.6 mm, 5 µm particle size). Elution was conducted at a flow rate of 0.5 mL/min with mixtures of 20 mM ammonium acetate (pH 7.5) in either H_2_O (solvent A) or acetonitrile (solvent B). Gradient elution was performed by increasing solvent A from 0 to 80% during a 30 min period, and then starting conditions were re-established in 1 min followed by re-equilibration for further 14 min. The detector operating parameters were: ion spray voltage: 4000 V; nitrogen flow: 12 L/min; fragmentation voltage: 100 V. EasyLCMS [29] was used for automated quantification. The quality of the results was assessed by (i) evaluation of the extraction method with standard mixtures; (ii) the use of an internal standard and (iii) quality control samples. The extraction method was validated by comparing the concentration of ppGpp standard mixtures before and after extraction, with ppGpp recoveries higher than 85% in all cases. The ppGpp standard was purchased from TriLink Biosciences. Thymine-methyl-d3-6-d (m/z 131) was added as internal standard (final concentration of 50 µM) to the samples to monitor the robustness of the analysis [30]. The acceptable coefficient of variation was set at 20% for the peak area and 2% for the retention time. Two types of quality controls were incorporated to the analysis including pools of samples and standards.

### Other analytical procedures

For the quantitative analyses of glycogen contents, the cells were grown in KM as described above, harvested at the indicated culture times, collected by centrifugation at 4400×g for 15 min, rinsed with fresh KM, resuspended in 40 mM Tris/HCl (pH 7.5), and disrupted by sonication as previously described [5], [7]. Quantitative glycogen measurement analyses were carried out in these extracts by using an amyloglucosidase based test kit (Boheringer Manheim). Protein content was measured by the Bradford method using a Bio-Rad prepared reagent, and β-galactosidase activity as described by Miller [31]. Qualitative analysis of glycogen content of cells cultured on solid KM was carried out using the iodine staining technique [22].

### Western blot analyses

The different BW25113 strains and derived Δ*spoT* clones analyzed in this work were cultured in liquid KM as described above and harvested after 7 h of culture. The cells were collected, rinsed, and subjected to sonic disruption as described previously [5], [7]. The bacterial extracts thus obtained were clarified by centrifugation (15000×g for 10 min), and total protein content was determined in the supernatants as described above. Soluble fractions corresponding to 70 µg of total proteins were subjected to SDS/PAGE (10% polyacrylamide gels), transferred to polyvinylidene difluoride filters (Bio-RAD, Hercules, CA, USA), and immunodetected by using anti-RelA mouse monoclonal antibodies (Santa Cruz Biotechnology, CA, USA) and goat anti-mouse IgG alkaline phosphatase conjugates. Immunoblots were developed with the 4-chloro-3-indolyl phosphate/nitroblue tetrazolium method. Alternatively, a goat anti-mouse IgG peroxidase conjugate (Sigma) was used as a secondary antibody and immunoblots were developed with Supersignal West Femto (Thermo scientific, IL, USA) followed by exposure to autoradiograph films.

### High-throughput whole genome sequencing

#### DNA fragment library preparations and pyrosequencing strategies

Bacterial DNA was extracted and purified by using the High Pure PCR Template Preparation Kit (Roche, Mannheim, Germany). Purified DNA was quantified by using a Quant-iT PicoGreen DNA quantification kit (Life Technologies) in a Bio-RAD VersaFluor Fluorometer. Depending on the sequencing strategy followed, the DNA samples were differentially treated as follows: For 454 pyrosequencing (Genome Sequencer FLX, LifeSciences, Roche Diagnostics, Branford, CT, USA) the samples were fragmented with the aid of a nitrogen nebulizer (400–800 nt length), followed by ligation to the sequencing adapters, emPCR, and sequencing following the manufacturer’s specifications. For Illumina sequencing (HiSeq 2000 system, Illumina Inc., San Diego, CA, USA), the DNA samples were fragmented with the aid of a Covaris S220 Focused-ultrasonicator (Covaris, Woburn, MA, USA) (250–700 nt length) followed by ligation to the adaptors and sequencing following the manufacturer’s specifications. The BW25113 genome was sequenced using both the 454 and Illumina platforms, whereas the rest of the strains were sequenced with the Illumina platform only.

#### Quality control, assembly and mapping of sequence reads

Only Illumina and 454 sequence reads with Q-values ≥30 and ≥25, respectively, were included in the analysis. All reads derived from the BW25113 (WT) strain were assembled using an incremental assembly procedure (Newbler version 2.6; 454 LifeSciences, Roche Diagnostics) with default parameters, except that the minimum length overlap and read length were set at 30 nt. 454 long reads were first assembled in a backbone where shorter Illumina reads were introduced and assembled. Assembly of the contigs corresponding to the WT strain was done using MAUVE [32] using as scaffold the genome of *E. coli* MG1655 (GenBank accession number NC_000913). The genome data obtained from the analyzed Δ*spoT* clones (5 and 10 to 13) were mapped using the gsMapper program version 2.6 (454 LifeSciences, Roche Diagnostics) with default parameters using the WT assembly data as reference to detect High Confidence Variations (HCDiffs) in the mutants.

#### Genome annotation and mutational profiling

All assembled contigs from the WT strain were introduced into the annotation pipeline implemented at Lifesequencing (Valencia, Spain). After searching the data for coding sequences, open reading frames and structural RNA (tRNA and rRNA) genes, the derived coding sequences and open reading frames (canonical and non-canonical) were associated to specific function(s) using BLASTX [33]. We also assigned GO terms and searched for any over-represented function taking into account annotated genes (http://www.geneontology.org/). Genes associated to a PFAM or Enzymatic code were also subjected to KEGG pathway mapping (http://www.kegg.jp/) [34]. Once the genome of the WT strain was annotated, HCDiffs mutations were mapped in each of the evolved strains and associated to specific genes. The number of HCDiffs mutations occurring in each gene was then calculated, and a mutational profiling was performed to allow the identification of genes in specific Δ*spoT* clones that accumulated more mutations than others. The genome sequence data of *E. coli* BW25113 WT and Δ*spoT* clones obtained in this work were deposited in the European Nucleotide Archive (EMBL-EBI) with the accession number PRJEB6520.

### Gene amplification and sequencing

*relA, dksA, rsd, ssrS, glnB*, *yejB, rpoB*, *rpoS, rpoC* and *rpoZ* genes and their immediate surrounding sequences, and sequences of the genomes of WT cells and Δ*spoT* clones that differed in the high-throughput sequencing analyses were amplified by PCR using the oligonucleotides described in **Table S2** (see also **Figure S2**). The amplicons thus obtained were subjected to Sanger sequencing at the Secugen S.L. DNA sequencing service (Madrid, Spain).

## Results and Discussion

### Correlation between glycogen accumulation and ppGpp levels in *E. coli* cells growing in KM

We selected KM for the experiments described in this work, reasoning that the use of enriched culture media for *E. coli* growth should allow a better definition of the threshold ppGpp cellular levels required to trigger glycogen accumulation. KM is composed of surplus glucose, yeast extract as a source of nitrogen compounds and vitamins, and a relatively high potassium phosphate concentration [21]. The glucose provides a surplus source of energy and carbon allowing steady growth to higher densities [35] and also net glycogen accumulation [22]. The yeast extract (0.6%) is the main source of most amino acids in both free and short peptides form [36], [37]. Finally, the relatively high potassium phosphate concentration (ca. 126 mM) is not only a surplus phosphate source but also helps maintaining the medium pH within limits compatible with cell growth [35].

**Figure 1:**
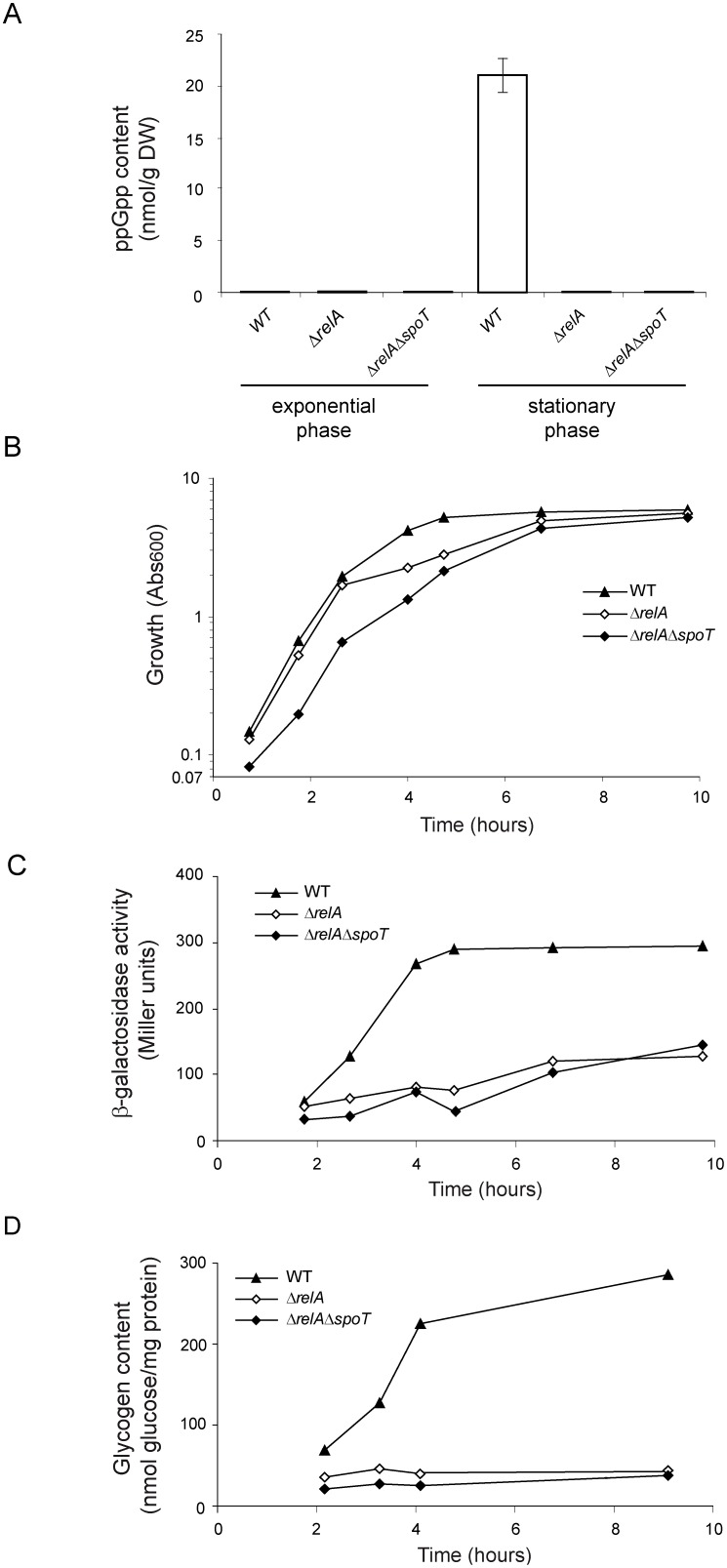
Growth, ppGpp and glycogen contents in BW25113 WT, Δ*relA* and Δ*relA*Δ*spoT* cells cultured in KM medium. (A) ppGpp content at the exponential and stationary phases, and time-courses of (B) growth, (C) chromosomal *glgB::lacZY* fusion expression, and (D) glycogen contents in WT cells, Δ*relA* cells and Δ*relA*Δ*spoT* cells. In “A”, cells at the exponential growth phase were harvested after 100 min of culture, whereas cells at the stationary phase were collected after 7 h of culture.

We first measured the intracellular ppGpp contents and carried out time course analyses of growth, glycogen content, and glycogen gene expression (as judged by the use of isogenic cells bearing the corresponding *glgB::lacZY* fusions) of WT, Δ*relA* and Δ*relA*Δ*spoT* (ppGpp^0^) cells in KM. As shown in **Figure 1A**, ppGpp contents in the WT strain at the exponential phase of growth were below detection limits (which were set at 0.6 nmol ppGpp/g DW for the HPLC/MS procedure used here). From this time period to the stationary phase the ppGpp levels in WT cells increased to around 22 nmoles ppGpp/g DW (**Figure 1A**). Expectedly, Δ*relA*Δ*spoT* double mutants showed no detectable ppGpp, a situation similar to Δ*relA* single mutants which also showed ppGpp levels below detection limits (**Figure 1A**). These results indicate that (a) RelA is the major source of ppGpp in *E. coli* cells growing in KM, and (b) the ppGpp synthetic activity of SpoT is largely impaired under these growth conditions, as judged by the absence of measurable ppGpp levels in Δ*relA* mutants. Since the RelA system serves the cell to monitor amino acid availability and ppGpp is rapidly degraded [9], the basal ppGpp levels found in WT cells in the stationary phase indicated substantial RelA activation and therefore that amino acid sources in KM were limiting during this phase. Experimental evidence indicated in fact that particular amino acids are sequentially depleted during *E. coli* growth in rich complex media [35], [38].

As seen in **Figure 1B**, growth of both the WT and Δ*relA* strains in KM occurred at similar rates during the first 2 h of growth (doubling times of 27±1 min and 28±1 min, respectively), reaching Abs_600_ values of 0.8–1.0. After this period growth began to slow down in these two strains with a more pronounced effect observed in the Δ*relA* mutant, although the final Abs_600_ at the stationary phase reached in both cases values of 4–5 (**Figure 1B**). As seen in the figure, growth of the Δ*relA*Δ*spoT* double mutant was slower than that of WT and Δ*relA* cells (doubling time of 37±3 min), although it finally reached Abs_600_ values of 4–5 after 7–10 h of growth (**Figure 1B**). It is worth noting that reductions in growth rates of Δ*relA*Δ*spoT* double mutants in other complex rich medium such as LB have been reported previously using different *E. coli* K-12 strains [15], [38]. In one of these studies [38] genetic evidence was additionally provided for the requirement of basal levels of ppGpp to induce an alternative pathway of synthesis of glutamine, an amino acid in short supply in either yeast extract- or peptone digest-based media [36], [38] and a required nitrogen donor for the synthesis of amino sugars, NAD, p-aminobenzoate, purines, pyrimidines, histidine, and tryptophan [39]. This could explain the differences in growth behavior between the WT strain and Δ*relA*Δ*spoT* double mutant in KM observed above. Moreover, the Δ*relA* single mutant seemingly adapted easier to this emerging metabolic necessity (**Figure 1B**), thus suggesting the synthesis of at least trace amounts of ppGpp as the result of SpoT action in these cells.

Concerning glycogen synthesis, it was observed for the WT strain that both glycogen gene expression (**Figure 1C**) and concomitant glycogen accumulation (**Figure 1D**) departed from basal levels after the culture surpassed the middle of the exponential phase, increased steadily during the onset of the stationary phase, and ceased abruptly as growth stopped. On the contrary, glycogen gene expression and glycogen accumulation in both Δ*relA*Δ*spoT* and Δ*relA* cells showed much lower increases above basal levels (**Figure 1C, D**). We infer from our data that whereas Δ*relA*Δ*spoT* cells accumulate no ppGpp, Δ*relA* cells could synthesize trace (but still below the detection limits of the HPLC/MS assay used here) levels of ppGpp as the result of SpoT action that were sufficient to stimulate growth but not glycogen gene expression in Δ*relA* cells.

The above results reinforced our previous observations that ppGpp represents a main positive regulator of glycogen gene expression and glycogen accumulation in *E. coli* [2], [5], [7]. Moreover, they also expanded our knowledge on the threshold ppGpp levels required to trigger net accumulation of this polysaccharide, which can be set at less than 22 nmoles ppGpp/g DW. It is worth noting that these values are in the range of the basal ppGpp levels determined by other authors for *E. coli* strains also growing in rich media supplemented with glucose, which ranged from ∼10 to 40 nmoles ppGpp/g DW [13], [40], [41]. Furthermore, these ppGpp levels are certainly much lower that those associated with the induction of RpoS-mediated stress responses in *E. coli* [17]. Our results also show that increases on glycogen gene expression and/or glycogen contents may provide reliable indicators of modifications of low basal levels of ppGpp associated to perturbations in steady state physiological growth.

### Production of Δ*spoT* cells

We next attempted the manipulation of the basal levels of ppGpp in *E. coli* by constructing mutants deficient in ppGpp degradation. All efforts to introduce a Δ*spoT::Spc* or a Δ*spoT::Km* allele into BW25113 *relA^+^* cells by the Datsenko and Wanner procedure [24] repeatedly failed in our hands. On the contrary, the transduction of the Δ*spoT::Spc* allele using P1 lysates of Δ*relA*Δ*spoT* cells into BW25113 (*relA^+^*) cells allowed us the selection of Δ*spoT::Spc* mutants as confirmed by both growth in Spectinomycin (Spc)-containing LB solid medium and the inability to amplify the WT *spoT* gene by PCR in these cells (see below). We also succeeded in transducing by the same P1-transduction procedure the Δ*spoT::Spc* null allele into other *E. coli* backgrounds including the B strains BL21 and B (not shown).

**Figure 2:**
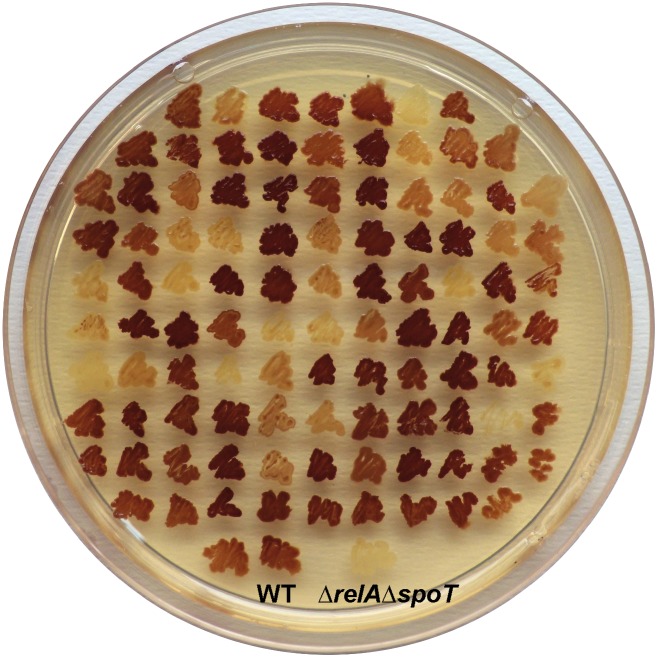
Glycogen iodine staining of Δ*spoT* clones. Iodine staining of BW25113 (WT), Δ*relA*Δ*spoT* double mutants, and 102 independent Δ*spoT* clones cultured in solid KM. In the presence of iodine vapors, “glycogen-excess” mutants stain darker than WT cells, whereas “glycogen-deficient” and “glycogen-less” mutants stain yellow.

The characterization by colony iodine staining [22] of 102 randomly-selected Spc-resistant transductants cultured in solid KM revealed substantial differences in glycogen content among them (**Figure 2**). As judged by visual inspection, approximately 18% of the colonies showed WT glycogen, 32% low glycogen and, unexpectedly, around 50% displayed high glycogen content phenotypes. Since ppGpp basal contents regulate glycogen gene expression and subsequent glycogen accumulation (see above), these results suggested wide differences in ppGpp basal contents among these Δ*spoT* clones. We thus selected 19 of them for further biochemical and genetic analysis: 8 with low glycogen (Δ*spoT* clones 1–8) and 11 with high glycogen (Δ*spoT* clones 9–19) content phenotypes (see **Table 1**). The selected 19 Δ*spoT* clones were cultured in Spc-containing medium and the presence of the Δ*spoT::Spc* deletion in each of these clones was confirmed by the inability to amplify the WT *spoT* gene by PCR (**Figure S1**).

### Characterization of Δ*spoT* clones in KM

**Figure 3:**
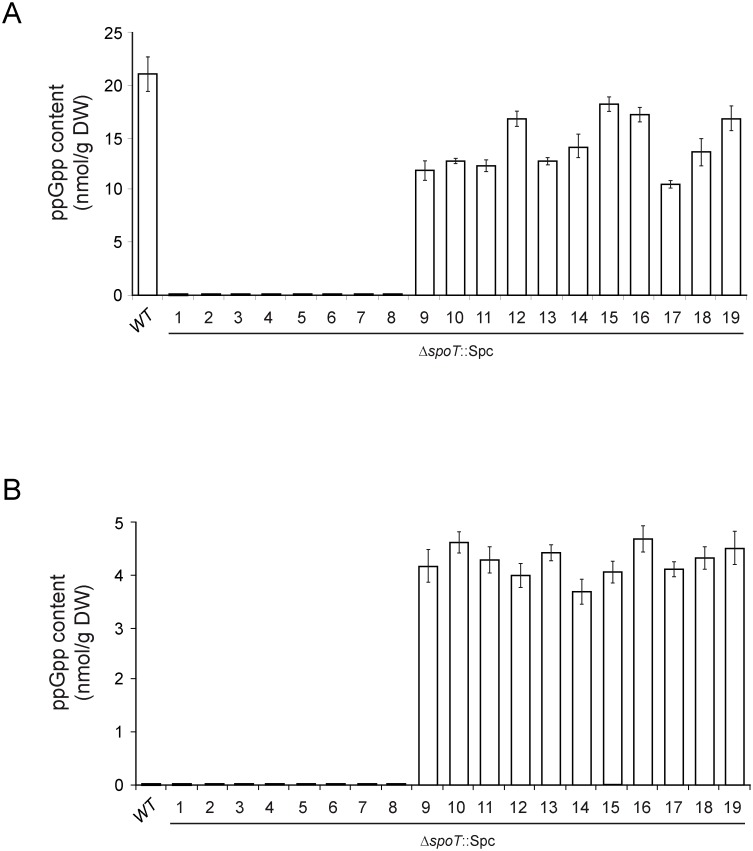
ppGpp contents in WT cells and in the 19 selected Δ*spoT* clones. Cells were cultured in liquid KM and harvested (A) at the stationary phase (after 7 h of culture) or (B) at the exponential growth phase (after 100 min of culture). The results are the mean ± SE of 3 independent experiments. Growth curves of WT and four representative Δ*spoT* clones are shown in **Figure 5A**.

**Figure 4:**
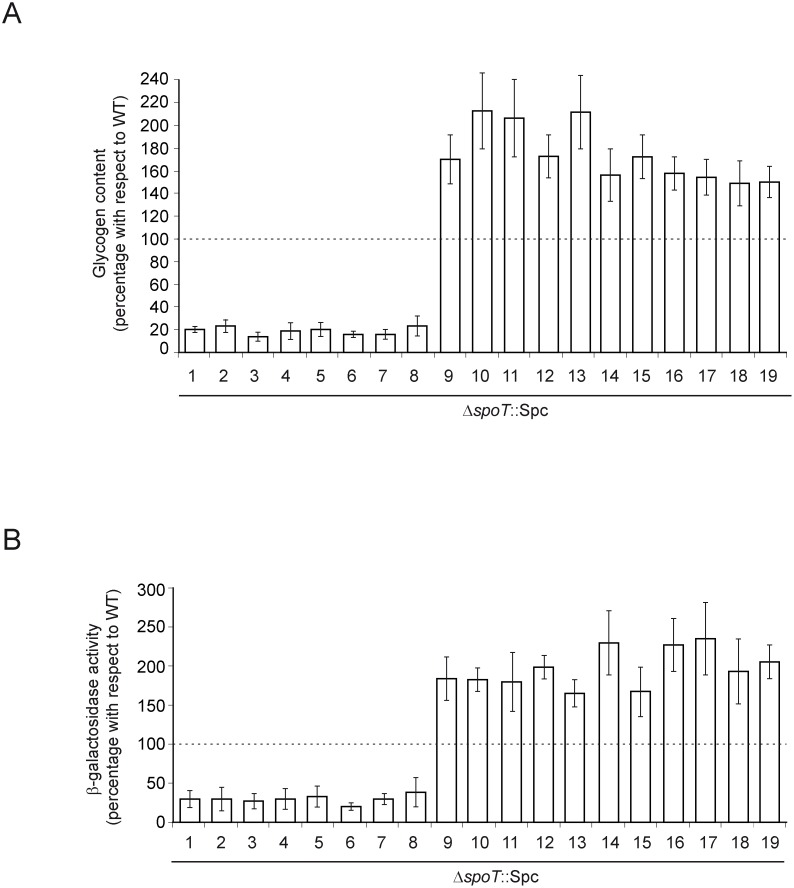
Glycogen contents and expression of chromosomal *glgB::lacZY* fusions in the 19 selected Δ*spoT* clones. Glycogen content and expression of chromosomal *glgB::lacZY* fusion at the stationary phase (after 7 h of culture) in WT cells, and in the 19 selected Δ*spoT* clones cultured in KM. Data are referred to as percentage of glycogen measured in WT cells. In “A”, average glycogen content in WT cells was equivalent to 257 nmol glucose/mg protein. In “B”, average level of *glgB::lacZY* expressions in WT cells was 312 Miller units [31]. The results are the mean ± SE of 3 independent experiments.

We next analyzed ppGpp and glycogen content, and glycogen gene expression at the stationary phase in the selected 19 Δ*spoT* clones grown in liquid KM. Δ*spoT* clones #1–8 showed ppGpp levels below detection limits at the stationary phase (**Figure 3A**). Expectedly, and similar to Δ*relA* or Δ*relA*Δ*spoT* mutants (cf. **Figure 1**), Δ*spoT* clones #1–8 exhibited both reduced glycogen gene expression and reduced glycogen content (**Figure 4**). In clear contrast, Δ*spoT* clones #9–19 exhibited 45–85% of the ppGpp contents found for the WT strain in the stationary phase (**Figure 3A**) and showed higher glycogen contents as the consequence of elevated glycogen gene expression levels (**Figure 4**).

**Figure 5:**
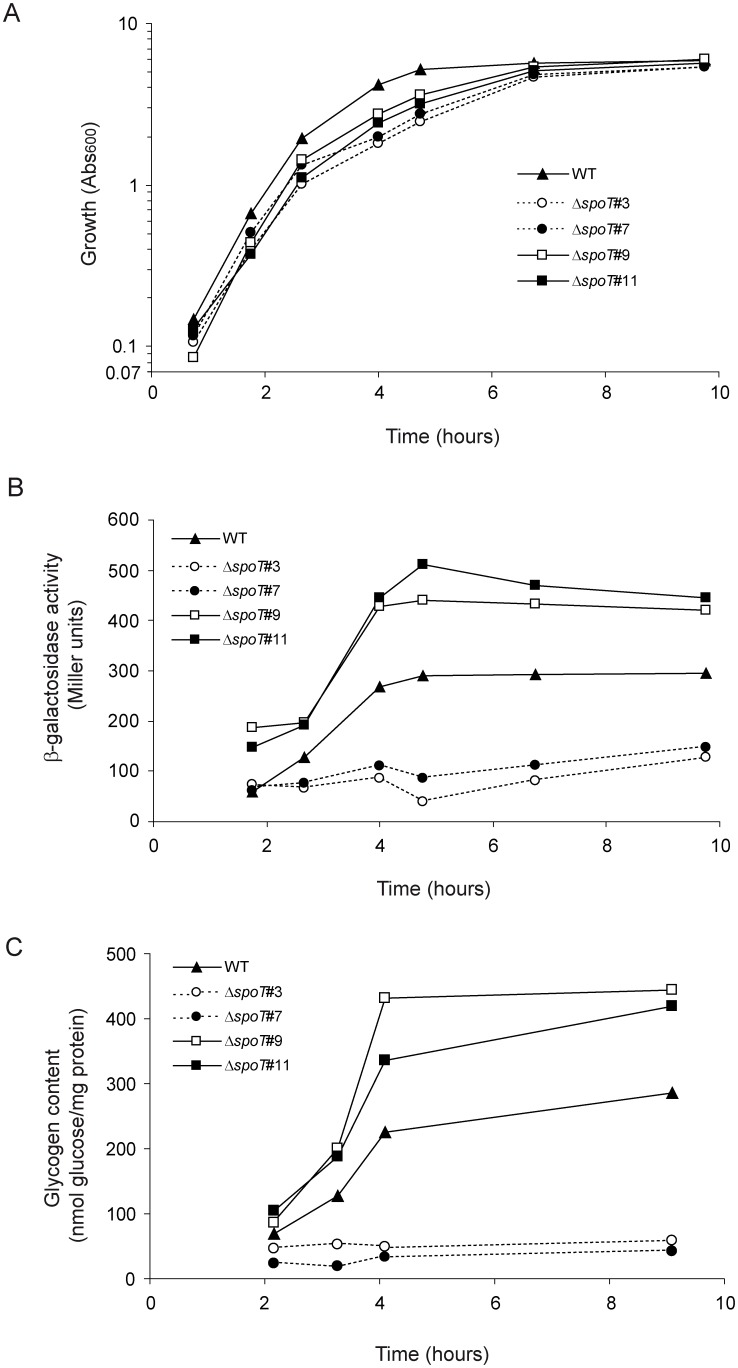
Kinetics of cell growth, glycogen accumulation, and expression of chromosomal *glgB::lacZY* fusions in representative ppGpp-less and ppGpp-containing Δ*spoT* clones cultured in liquid KM. Time-course of (A) cell growth, (B) expression of chromosomal *glgB::lacZY* fusions, and (C) glycogen accumulation of WT cells and representative Δ*spoT* clones displaying either no detectable ppGpp (clones 3 and 7) or significant ppGpp accumulation (clones 9 and 11).

We also conducted time-course analyses of growth, expression of glycogen genes, and glycogen content of the 19 selected Δ*spoT* clones cultured in KM. Two representative examples of Δ*spoT* clones with low glycogen content (clones #3 and #7) and two with high glycogen content (clones #9 and #11) are shown to illustrate the results (**Figure 5**). These analyses indicated that growth behavior of Δ*spoT* clones #1–8 was comparable to that of Δ*relA* single mutant (cf. **Figure 1B**). That is, growth proceeded exponentially for a period of about 2 h with calculated doubling times of 30±2 min to Abs_600_ values of around 0.6–0.8, and then slowed down, finally reaching Abs_600_ values of 4–5 after 6–7 h (**Figure 5A**). Similar to the situation found at the stationary phase (cf. **Figure 3A**), ppGpp contents were below detectable limits at the exponential phase in Δ*spoT* clones #1–8 (**Figure 3B**), thus explaining their largely reduced or null induction of glycogen gene expression and glycogen accumulation during growth (**Figures 5B and 5C**). For Δ*spoT* clones #9–19 growth behavior was comparable to that of Δ*spoT* clones #1–8, being exponential in all cases for about 2 h with doubling times of 29±2 min to Abs_600_ values of around 0.7–0.9, and then slowed down reaching Abs_600_ values of 4–5 after 6–7 h (**Figure 5A**). These clones, however, showed time-course profiles of glycogen gene expression and concomitant glycogen content similar to those of the WT strain except that at higher values at any equivalent time (**Figures 5B and 5C**). These results suggest the presence of basal levels of ppGpp significantly higher than those of WT cells during exponential growth. In line with this presumption, an average of ca. 4 nmoles ppGpp/g DW was detected in Δ*spoT* clones #9–19 after 100 min of growth in KM (**Figure 3B**). The premature rise of basal ppGpp levels in these clones did not affect growth to a significant extent as judged by the similar growth curve profiles obtained for Δ*spoT* clones #9–19 and #1–8 (**Figure 5A** and data not shown).

The results obtained so far indicated that the introduction of a Δ*spoT* null allele into *relA* ^+^ cells of *E. coli* resulted not only in the (expected) selection of *relA* mutants lacking ppGpp synthesis capability, but also in a significant percentage of clones (Δ*spoT* clones #9–19) bearing *relA* alleles containing non-inactivating mutations as judged by the measurable levels of ppGpp at the exponential phase of growth in KM (in average, 4 nmoles ppGpp/g DW, cf. **Figure 3B**). This, in turn, resulted in concomitant induction of glycogen gene expression and glycogen accumulation well above basal levels. Moreover, additional increases of basal ppGpp levels were observed in these particular clones as cells entered into the stationary phase, as judged both by ppGpp determinations after 7 h of growth (average of 14 nmoles ppGpp/g DW, cf. **Figure 3A**) and further inductions of glycogen gene expression and glycogen accumulation (**Figures 5B and 5C**). These results are compatible with the presence in Δ*spoT* clones #9–19 of mutant RelA proteins variants retaining both enzymatic capability and inducible properties as growth proceeded and nutrient sources became exhausted. They also pose the question on whether all the *relA* suppressor alleles selected in these Δ*spoT* clones code for RelA variants displaying increased basal levels of ppGpp-synthesizing activity, or the WT RelA itself displays low basal ppGpp synthesizing activity in cells that are not nutritionally limited which could be exposed only by the complete removal of *spoT* as reported here. The latter alternative could also explain the question as to why ppGpp accumulates at all in cells that are not limited for amino acids or aminoacyl-tRNA when RelA is overproduced from plasmids [9].

Another important conclusion derived from the analysis of Δ*spoT* clones #9–19 concerns the threshold ppGpp levels that suffice to induce glycogen gene expression and concomitant glycogen accumulation in *E. coli*, which can be set now in around 4 nmoles ppGpp/g DW as judged from the above results.

### Characterization of ΔspoT clones in M9-glucose minimal medium

**Figure 6:**
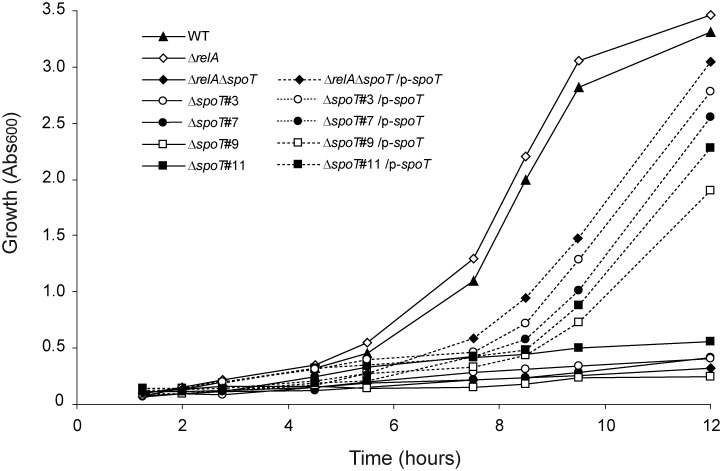
Growth behavior of Δ*relA* single mutants, Δ*relA*Δ*spoT* double mutants, and four representative Δ*spoT* clones cultured in M9-glucose medium. Growth in glucose-containing M9 minimal medium of Δ*relA* and Δ*relA*Δ*spoT* mutants as well as of representative ppGpp-less (3 and 7) and ppGpp-containing (9 and 11) Δ*spoT* clones. All Δ*spoT* cells tested were transformed with the empty plasmid vector pCA24N-EV or with pCA24N-*spoT* expressing the cloned *spoT* gene (p-*spoT*) as indicated in the inset. Aliquots were taken at the indicated times for Abs_600_ measurements. For details see Materials and Methods.

Likely due to the multiple amino acid auxotrophies resulting from their ppGpp^0^ status, *E*. *coli* Δ*relA*Δ*spoT* double mutants are generally unable to grow in minimal media containing glucose and ammonia as carbon and nitrogen sources, respectively [9], [20]. On the contrary single Δ*relA* mutants are capable of growing in such media, a situation attributed to the SpoT-mediated synthesis of ppGpp to levels promoting expression of amino acid and other biosynthetic operons [9], [20], [42]. In agreement, BW25113Δ*relA*Δ*spoT* double mutants were unable to grow in M9-glucose minimal medium, a phenotype which could be reverted by the expression of *spoT* from plasmids (**Figure 6**). Also, BW25113Δ*relA* single mutants displayed a growth behavior similar to that of WT cells thus reinforcing the role of SpoT as a source of ppGpp required for growth in M9-glucose.

Considering the different ppGpp synthesizing capabilities of the two different groups of Δ*spoT* clones described above, we thus found of interest to compare their growth behavior in M9-glucose (**Figure 6**). Similar to Δ*relA*Δ*spoT* double mutants, Δ*spoT* clones #1–8 lacking detectable ppGpp production showed severe grow impairments in minimal medium (**Figure 6** and data not shown). This represented an expected behavior considering the lack of detection of ppGpp in these cells (**Figure 3**), suggesting either null or very low production of this nucleotide to levels still incapable to activate biosynthetic operons necessary for growth in minimal medium. In agreement, the growth impairments shown by Δ*spoT* clones #1–8 was reverted by the introduction of a *spoT*-expressing plasmid in the cells (**Figure 6**). This reinforced the above notion of essential functions of SpoT for the growth of these cells in minimal medium. Remarkably, Δ*spoT* clones #9–19 (which showed the capability to accumulate significant amounts of ppGpp in KM, see above) were also severely impaired to grow in M9-glucose (**Figure 6**). Moreover, the growth blockage phenotype of these Δ*spoT* clones could also be reverted by the expression of *spoT* in *trans* from plasmids (**Figure 6**).

The above observations can be explained in many ways. The combined evidence of the growth behavior phenotypes of WT, Δ*relA*, Δ*relA*Δ*spoT*, and Δ*spoT* clones #1–8 in M9-glucose, including the fact that *spoT* complementation in *trans* restored growth of both Δ*relA*Δ*spoT* double mutants and Δ*spoT* clones in this medium, supports the notion that SpoT can provide the ppGpp basal levels required for *E. coli* to initiate growth under these conditions. The lack of growth of Δ*spoT* clones #9–19 (all retaining substantial ppGpp synthesis capability, see above) could be explained by assuming that a rapid burst of RelA-mediated ppGpp accumulation reaching levels impairing growth occurred when these cells were transferred to M9-glucose. In such a case, the provision of *spoT* in *trans* would reinstate the ppGpp hydrolytic activity necessary to prevent excessive ppGpp accumulation, thus allowing growth to proceed [20], [42]. Another possibility, which is not mutually exclusive with the others is that, concomitant to its participation in ppGpp metabolism, SpoT plays other function(s) necessary for *E. coli* growth in M9-glucose. In this context SpoT has been shown to physically interact with essential proteins such as the acyl carrier protein involved in fatty acid biosynthesis, an interaction related to the activation of the SpoT ppGpp synthase activity in conditions of fatty acid starvation [42]. SpoT has also been shown to interact with ObgE/CgtA, a weak GTPase involved in cell division, ribosome functions, and regulation of the pppGpp/ppGpp cellular ratio during amino acid starvation [43], [44]. The biological significance of this interaction remains poorly defined, but the possibility of other essential roles of SpoT for *E. coli* growth under minimal conditions deserves further studies.

### High levels of genetic polymorphism at the *relA* locus among the selected Δ*spoT* clones

**Figure 7:**
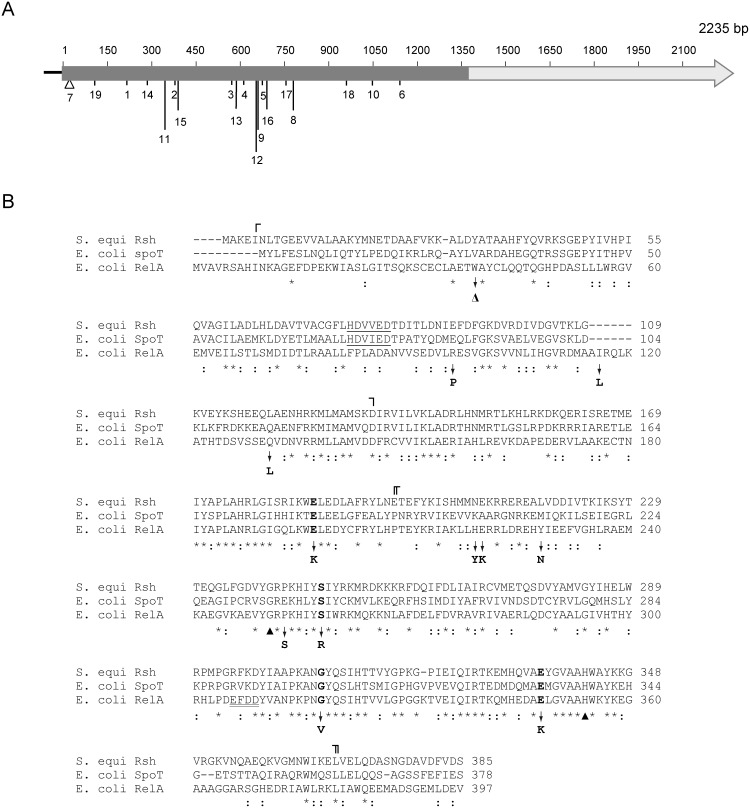
Schematic representation and sequence characterization of the different *relA* mutations identified in the Δ*spoT* clones studied here. A. The designation of each Δ*spoT* clone characterized in this work is indicated below the *E. coli relA* gene representation. The RelA region encoding the N-terminal 455 amino acid residues bearing ppGpp synthetase activity [9], [45] is shadowed. B. Amino acid sequence comparisons of the N-terminal regions (∼400 amino acid residues) of *S. equisimilis* Rsh, *E. coli* RelA, and SpoT. The alignments of the corresponding regions were constructed by using ClustalW (http://www.genome.jp/tools/clustalw) using default parameters, and subsequently refined to maximize secondary structure similarities between *S. equisimilis* Rsh [46] and RelA (as predicted by Jpred3; http://www.compbio.dundee.ac.uk). The corresponding amino acid sequence positions are indicated at the right. Identical (*) or conserved (:) amino acids at a given position among the three sequences are indicated below the alignments, and deletions/insertions by hyphens (−). The sequence regions spanning the ppGpp hydrolase (⌜ ⌝) and synthetase (⌜ ⌝) domains of *S. equisimilis* Rsh, as well as the central-3 helix bundle region joining these domains defined by the ⌝ and 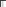 boundaries [46] are indicated above the alignments. The different amino acid substitutions and the Trp39 deletion (Δ) identified in *E. coli* RelA in Δ*spoT* clones 8–19 (this work) are indicated below the alignments. Conservation of equivalent residues in *S. equisimilis* Rsh and *E. coli* SpoT is indicated by highlighting the corresponding amino acids in bold. Conserved residues whose site-directed mutagenesis has been found by other authors to promote loss (G251E) or reductions (H354Y) in *E. coli* RelA ppGpp synthesis capability [45] are indicated by closed arrowheads below the alignments. The HDXXED motif typical of metal-dependent pyrophosphohydrolases located in the hydrolase domain of bifunctional enzymes [46], [55] is underlined. In turn, the synthetase domain motif distinguishing monofunctional (EXDD) from bifunctional (RFKD) enzymes [46], [55], [56] is double underlined. The sources of the sequences were: *S. equisimilis* Rsh, ref. 46; *E. coli* K-12 MG1655 SpoT, GenBank accession NP_418107.1; *E. coli* RelA BW25113: this work. The latter is identical to *E. coli* K-12 MG1655 RelA, GenBank accession NP_417264.1.

**Figure 8:**
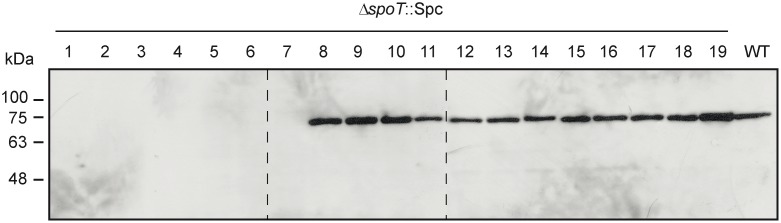
Western blot analysis of RelA in WT, and in the 19 selected Δ*spoT* clones. The gel was loaded with 70 µg per lane of total soluble proteins of each of the described cells. The different bacterial strains were cultured in liquid KM and harvested after 7 h of culture. This figure is a composite of 3 different western blot scans, whose separation is indicated by dotted lines.

Reasoning that survival of Δ*spoT* mutants could be ascribed to mutations in *relA*, we sequenced the *relA* in all of the 19 selected Δ*spoT* clones. As shown in **Table 1** and **Figure 7**, these analyses revealed different inactivating mutations at the *relA* locus in Δ*spoT* clones 1 to 8 including: i) Nonsense mutations in Δ*spoT* clones #1–6 introducing premature stop codons at different positions within the coding region of the 455 amino acid residues N-terminal domain possessing the ppGpp synthetase catalytic site [9], [45]. In the resulting truncated polypeptides this catalytic region is either completely removed or severely reduced in length depending on the clone analyzed (**Figure 7A**); ii) an IS1 insertion sequence immediately downstream of the *relA* initiation codon in Δ*spoT* clone #7, and; iii) a missense mutation in Δ*spoT* clone #8 resulting in the replacement of a Ser residue located at position 258 for an Arg (S258R) (**Table 1**, see also **Figure 7**). In agreement with the *relA* sequencing results and the absence of ppGpp, immunoblot analyses indicated no detectable RelA proteins in Δ*spoT* clones #1–7 (**Figure 8**). In the case of Δ*spoT* clone #8, the S258R replacement highly impaired ppGpp synthesis capability as judged by the lack of detection of ppGpp in this mutant (**Figure 3**). Still, this mutation allowed the synthesis of a full-length, metabolically stable RelA protein, as judged by its presence in the soluble fractions of cell extracts (**Figure 8**). It is worth noting that replacement of the equivalent Ser residue located in the catalytic loop of the RelA/SpoT (Rsh) bifunctional enzyme of *Streptococcus equisimilis* (Ser247, **Figure 7B**) also impaired ppGpp synthesis [46]. The combined observations described above provide a reasonable explanation for the lack of detection of ppGpp in Δ*spoT* clones #1–8.

For Δ*spoT* clones #9 to #19 sequence analysis indicated the presence of missense mutations in *relA* located at different positions all along the N-terminal coding region of RelA (clones #9–18) and a deletion of a nucleotide triplet (clone #19) corresponding to Trp39 (**Table 1** and **Figure 7**). In all of these clones the presence of RelA in the corresponding soluble cell fractions was detected by immunoblot analysis (**Figure 8**). This, in addition to the substantial ppGpp accumulation (**Figure 3**), indicated that metabolically stable and catalytically competent RelA variants were produced in all of these Δ*spoT* clones. Expectedly, many of the amino acid changes were located either in the close immediate region (E197K) or within (H219Y, Y228N, P253S, G318V, and E351K) the ppGpp synthetase homolog domain of RelA (**Figure 7B**). This supports the idea that these particular RelA mutations could account for the reductions in ppGpp accumulation described above for these Δ*spoT* clones. Most remarkably, other changes also associated to reductions in ppGpp accumulation such as the Trp39 deletion and the R96P, I116L, and Q131L replacements are located in the N-terminal region of RelA displaying substantial homology to the hydrolase domain of RelA/SpoT bifunctional enzymes (**Figure 7B**).

### Sequence analysis of ppGpp-synthesizing Δ*spoT* clones

We next analyzed the possibility that mutations in other gene(s) could be co-selected with those described above in *relA* to help compensating the deleterious effects of SpoT loss in the above Δ*spoT* clones. In this context suppressor mutations in *rpoB* have been found to relieve the toxic effects of high cellular levels of ppGpp [9], [47]. High ppGpp strains have been also found to accumulate mutations in *rpoS* [48]. The *rpoZ* gene product has been recently shown to be crucial for ppGpp-RNA polymerase interactions, and could therefore provide a source of suppressor mutations [49], [50]. Mutations at the *rplK* (formerly *relC*) locus coding for ribosomal protein L11 involved in RelA activation [9] could have also been selected in cells overproducing ppGpp. In turn, ppGpp-deficient strains have been found to accumulate suppressor mutations in *rpoB*, *rpoC*, or *rpoD* [9]. Mutations in genes coding for mediators or targets of ppGpp actions such as *dksA*, *rsd*, or *ssrS* [9]–[12] could have also been selected to compensate altered cellular ppGpp levels. However, Sanger sequence analysis of *rpoB*, *rpoS*, *rsd, ssrS, rpoC, rpoZ* and *dksA* in all the 19 Δ*spoT* clones studied here revealed in all cases identical sequences to the corresponding genes in the WT strain (**Table 1**). Moreover, high-throughput genome sequence analysis of five selected Δ*spoT* clones (clones 5, 10, 11, 12 and 13, see **Table 2**) not only confirmed the above Sanger sequence analyses, but also failed to identify mutations at the *rplK* or *rpoD* loci that could have been co-selected with the *relA* mutations present in these clones. Furthermore, the whole genome sequencing of the five selected Δ*spoT* clones revealed that the mutations are mainly restricted to *relA.* As shown in **Table 2**, three of these clones showed only mutations in *relA* in their genomes including a premature stop codon in Δ*spoT* clone #5, and missense changes in *relA* in Δ*spoT* clones #11 and #12. In turn, and besides missense changes in *relA*, additional mutations were detected in *yejB* and *glnB* (Δ*spoT* clones #10 and #13, respectively), which have not been characterized as genetically linked to *relA* [9].

**Table 2:** Description of mutations identified by whole genome sequencing of five Δ*spoT* clones (5, 10, 11, 12 and 13).

Gene	Δ*spoT* clone	Ecogene description	Mutation	Effect
*relA*	5, 10, 11, 12, 13	ppGpp synthase I; ppGppsynthesis during stringent response	See **Table 1**	See **Table 1**
*glnB*	13	PII regulatory proteinfor glutamine synthase	Deletion of 3 nt (ATG)between positions 82–84of the gene	Deletion of Met28
*yejB*	10	Microcin C transporter YejABEF, permeasesubunit; ABC family	G for T change at ntposition 142	Production of a truncated polypeptide of 47 amino acids

The observation that only few mutated loci other than *relA* are present in the genomes of the *E. coli* Δ*spoT* clones analyzed here is noteworthy, especially when compared to other bacteria such as the Gram positive species *Bacillus subtilis* in which more changes were identified in their genomes accompanying the mutations compensating the lack of the RelA/SpoT bifunctional enzyme present in these organisms [51]. The overall results shown above thus reinforced the notion that the mutations in *relA* described in this work represent *bona fide* suppressors of total *spoT* loss in *E. coli*, and that *relA* mutations are predominantly selected to compensate the detrimental effects of SpoT mutational loss in this bacterial species [9].

### Additional and concluding remarks

The aim of this work was to better understand the details of ppGpp control on *E. coli* glycogen metabolism. The results of this work reinforced the role of ppGpp as a main positive regulator of glycogen gene expression and glycogen accumulation in *E. coli* [2], [5], [7]. Moreover, they expanded our knowledge on the threshold basal levels of ppGpp required to trigger net glycogen accumulation in *E. coli* K-12, which were estimated here in around 4 nmoles ppGpp/g DW by the use of a highly sensitive MS/HPLC procedure. These relatively low values are more compatible with ppGpp cellular levels related to the induction of amino acid biosynthetic operons as external sources are exhausted than to the much higher values associated with severe growth impairments and the induction of RpoS-mediated stress responses [17], [18].

In the course of our studies we constructed and characterized a series of *spoT* null mutants of *E. coli* K-12 which were obtained by transducing a Δ*spoT* null allele from Δ*relA*Δ*spoT* cells into *relA^+^* receptor bacteria followed by selection in LB rich medium. Different sequencing approaches conducted here on distinct Δ*spoT* clones confirmed that *relA* represents the primary source of suppressor mutations compensating the deleterious effects of SpoT loss in *E. coli*. Expectedly, different inactivating mutations were found in *relA* such as those present in Δ*spoT* clones #1–8 which lacked detectable ppGpp production and thus both glycogen gene expression and accumulation (**Figures 3** **,** **4** **,** **5**). Remarkably however, around half of the Δ*spoT* mutants obtained after transduction (clones #9–19) contained missense mutations at the *relA* region encoding the N-terminal catalytic domain that generated stable, full-length RelA variants retaining substantial ppGpp synthesizing capability. These RelA variants showed altered regulation as showed by the significant basal levels of ppGpp found in these cells as compared to WT cells during exponential growth in KM. Yet, and similar to WT RelA, these mutant variants were also activated as cell growth proceeded into the stationary phase as judged by both ppGpp determinations and steady increases on both glycogen gene expression and accumulation (**Figures 3** **,** **4** **,** **5**).

To our knowledge this is the first report describing the systematic production of *spoT* null mutants of *E. coli* in which *relA* suppressor alleles encoding enzymes retaining both significant ppGpp synthesizing and regulatory capabilities have been selected. Results presented in this work opened questions on how RelA activity is controlled in Δ*spoT* clones 9–19 during growth in KM within limits compatible with optimal growth. The absence of ppGpp over-accumulation in these clones in the stationary phase could be ascribed to altered kinetic properties of the mutant RelA proteins, or alternatively to the occurrence of ppGpp degradative enzymes that help preventing the building up of toxic ppGpp levels. In this respect we must emphasize that, although it is generally assumed that SpoT represents the main *E. coli* ppGpp degradative enzyme [9]–[12], other poorly characterized activities may also play role(s) in preventing ppGpp over-accumulation [9], [52]–[54].

Both RelA and SpoT form part of a sequence-related superfamily of enzymes (RelA/SpoT or Rsh) of around 700–800 amino acid residues present in both bacteria and plants, which are devoted to (p)ppGpp synthesis and breakdown [9]–[12]. Many members of this superfamily, such as the enzymes present in many Gram-positive bacteria and *E. coli* SpoT itself, are bifunctional enzymes endowed with both (p)ppGpp synthetase and hydrolase activity [9]–[12]. *E. coli* RelA and its close sequence homologs are considered specialized monofunctional ppGpp synthetases generally lacking hydrolase activity [9]–[12], [45], [55], [56]. Both bifunctional and monofunctional enzymes are composed by an N-terminal region bearing the catalytic site(s), and a C-terminal region responsible of regulatory functions on the N-terminal domain derived from its interaction with the ribosome and other effectors, oligomerization, etc. [9]–[12], [45], [46], [54]. The synthetase and hydrolase domains of the N-terminal region of the Rsh bifunctional enzyme from *Streptococcus equisimilis* were found to reside in separate domains within the first 385 amino acid residues of this protein [46] (**Figure 7B**). Evidence for cross-talk/reciprocal regulation between these two antagonistic catalytic domains has been derived from different structural and mutagenesis analyses [10], [46], [54]. In addition, the amino acid sequence homology existing between the N-terminal regions of RelA, SpoT and Rsh proteins (**Figure 7B**) suggests that, besides some functional differences among these enzymes, all share similar ppGpp synthetase catalytic domains [9]–[12]. Previous site-directed mutagenesis studies of conserved residues located in the proposed synthetase domain of *E. coli* RelA that resulted in either loss (G251E) or reductions (H354Y) of ppGpp synthesis capability also supported this notion [45]. However, since these findings, a number of differences have been found between bifunctional and monofunctional enzymes, such as a charge reversal in a motif located in the synthetase domain (**Figure 7B**) (which may account for differential metal-mediated catalytic mechanisms), GTP/GDP substrate specificity, cooperativity, regulation of the N-terminal catalytic activities by the C-terminal region, and even a new catalytic activity [55], [56]. Also, the sequence homology detected between bifunctional enzymes and RelA at the level of the hydrolase domain (**Figure 7B**) raised other questions such as whether the latter may have residual or cryptic ppGpp hydrolase activity [9]. Although RelA lacks the conserved HDXXED motif typical of metal-dependent phosphohydrolases [46], [55], [56], the possibility still exists that such activity could be invoked by certain amino acid substitutions. Other evolutionary pressures could alternatively be involved in the preservation of a hydrolase-like domain in monofunctional enzymes, which may be more related to the regulation of the nearby ppGpp synthetase domain similar to the case of bifunctional enzymes discussed above.

Many of the questions posed above can be addressed by the relatively straightforward *relA* mutant generation procedure described here. In fact, and as proof of concept, it served to support an essential function of Ser258 for *E. coli* RelA ppGpp synthetase activity (this work). In *S. equisimilis* Rsh the equivalent Ser residue (Ser247) forms part of the α13 helix of the α13/β4 ppGpp synthetase catalytic loop, and mutational replacements of this residue impaired ppGpp synthesis [46]. The overall observations thus suggest similarities between the catalytic mechanisms operating in monofunctional and bifunctional enzymes.

The procedure reported here was useful to identify novel amino acid residues in *E. coli* RelA whose mutational replacements resulted in reductions of ppGpp synthesis capability such as His219, Glu220, Tyr228, Pro253, Gly318, and Glu351, all located in the synthetase domain of this enzyme (**Figure 7**). Notably, however, all replacements found at these residues apparently reduced but not abolished RelA ppGpp synthesis capability, an observation that certainly deserves further studies. A similar situation was found for Glu197, a conserved residue located in the region spanning between the hydrolase-like and synthetase domains of RelA (**Figure 7**). It is worth remarking that all mutations found in the equivalent central-3 helix bundle region of *S. equisimilis* Rsh impaired ppGpp hydrolase rather than synthetase activity [46]. These observations suggest that differences also exist between monofunctional and bifunctional enzymes which are originated in sequence regions distinct from the charge reversal motif recently described [55], [56]. Most remarkably, amino acid mutations in the hydrolase homolog domain of RelA such as the deletion of Trp39 and the replacements of residues Arg96, Gln131, and Ile116 (which is located in a 6-residue stretch that is missing in bifunctional enzymes) (**Figure 7B**) were all found to reduce ppGpp synthesis capability. This suggests that reciprocal regulation between these two separate N-terminal domains also exists in *E. coli* RelA, a situation that would provide a reasonable explanation for the overall sequence conservation found all along this region between monofunctional and bifunctional enzymes (**Figure 7B**). In addition, it helped identifying some amino acid residues potentially involved in regulatory actions interconnecting both RelA domains.

The procedure described here thus provides a useful tool for a better understanding of the structure/function relationships of RelA monofunctional members of the Rsh family. Yet, ppGpp plays important roles in aspects beyond glycogen metabolism including the activation of the Lrp regulon and thus amino acid biosynthetic operons, catabolite de-repression, DNA synthesis, rRNA and tRNA synthesis, fatty acid metabolism, stress responses, surface organelle and biofilm production, pathogenesis, etc. [9]–[12], [17], [18]. Therefore, the series of mutants obtained in this work, and the simple glycogen iodine-staining based method useful for the identification of *relA* suppressor mutations in a variety of bacterial backgrounds, may help future studies of ppGpp-mediated regulation of the above biological processes under different scenarios.

## Supporting Information

Figure S1 **Production of Δ** ***spoT*** **BW25113 cells.** The presence of the Δ*spoT::Spc* deletion allele in the 19 selected Spc-resistant clones was confirmed by PCR using the O1 and O2 primers flanking the site of the mutation (upper panel) and by the inability to amplify *spoT* by PCR using the *spoT* specific internal primers O3 and O4 (lower panel) (**Table S2**). The same results were obtained using other K-12 and B Δ*spoT::Spc* strains constructed in this work (not shown). The figure represents a composite of two different gels, whose separation is indicated by dotted lines.
(TIF)

Figure S2 **Scheme illustrating the location of the different primers described in Table S2 used for PCR amplification of the different** ***E. coli loci*** **analyzed in this study.** The different *loci* analyzed and the expected hybridization regions of the corresponding sets of forward and reverse primers are indicated above each of the figures. The length of each of the corresponding genes and their neighboring regions (in bp) are also indicated.
(TIF)

Table S1 **Oligonucleotides used for PCR constructions employed in the deletion of the** ***E. coli spoT*** **gene.** *spoT* deletion was done following the method of Datsenko and Wanner [24]. Priming sequences for the Spc resistance gene are indicated in bold.
(DOC)

Table S2 **Oligonucleotide primers used for PCR amplification of the different** ***E. coli loci*** **analyzed in this study.** For further details see **Figure S2**.
(DOC)
