# Supplementary figures and images for: Systematic Production of Inactivating and Non-Inactivating Suppressor Mutations at the relA Locus That Compensate the Detrimental Effects of Complete spoT Loss and Affect Glycogen Content in Escherichia coli
Source: PLoS One. 2014 Sep 4;9(9):e106938. doi: 10.1371/journal.pone.0106938 (PMC4154780; doi:10.1371/journal.pone.0106938)

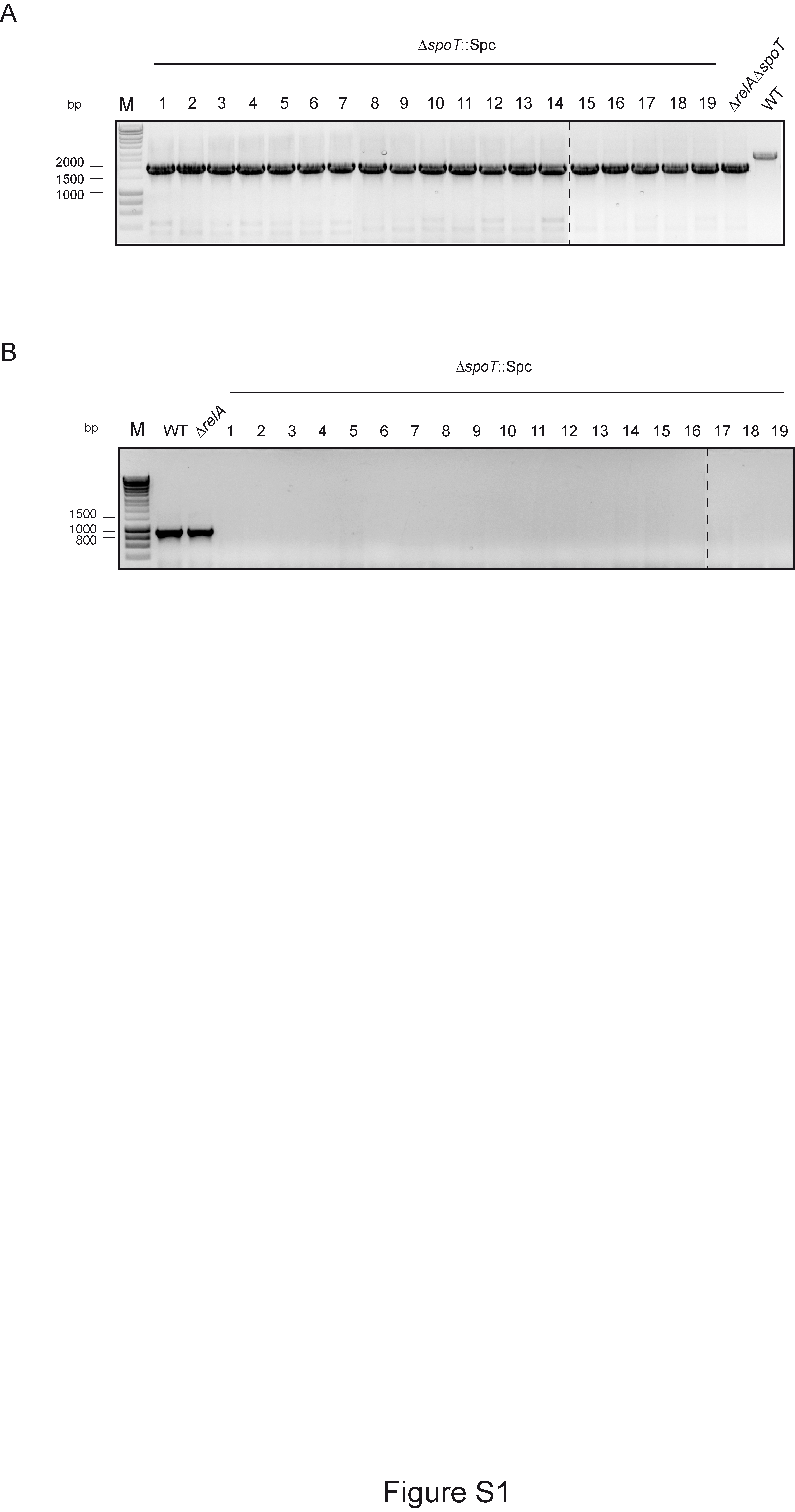

Supplement: Figure S1 — Production of Δ spoT BW25113 cells. The presence of the ΔspoT::Spc deletion allele in the 19 selected Spc-resistant clones was confirmed by PCR using the O1 and O2 primers flanking the site of the mutation (upper panel) and by the inability to amplify spoT by PCR using the spoT specific internal primers O3 and O4 (lower panel) (Table S2). The same results were obtained using other K-12 and B ΔspoT::Spc strains constructed in this work (not shown). The figure represents a composite of two different gels, whose separation is indicated by dotted lines. (TIF) [file pone.0106938.s001.tif]

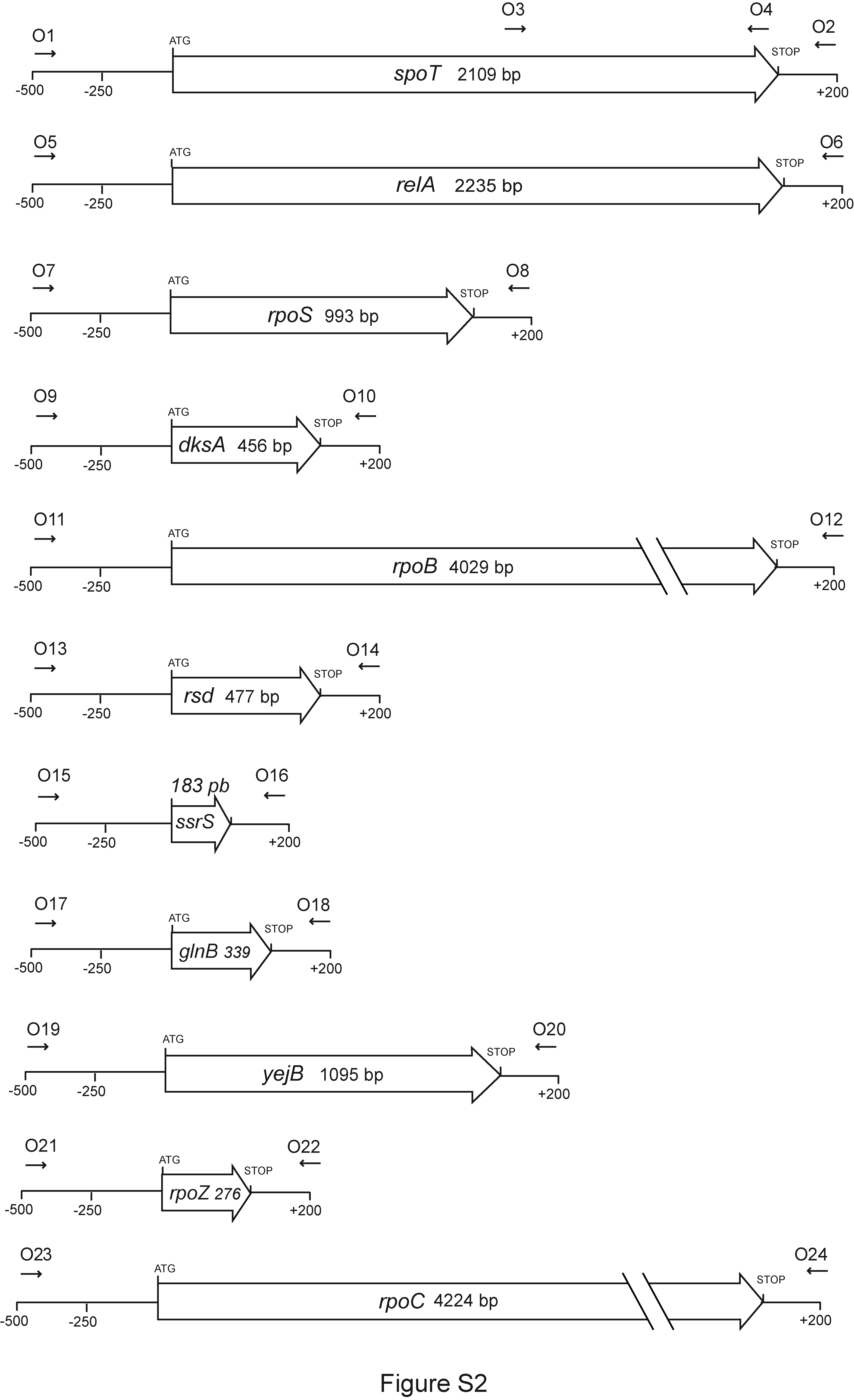

Supplement: Figure S2 — Scheme illustrating the location of the different primers described in Table S2 used for PCR amplification of the different E. coli loci analyzed in this study. The different loci analyzed and the expected hybridization regions of the corresponding sets of forward and reverse primers are indicated above each of the figures. The length of each of the corresponding genes and their neighboring regions (in bp) are also indicated. (TIF) [file pone.0106938.s002.tif]
