# Supplementary material for: Systematic Production of Inactivating and Non-Inactivating Suppressor Mutations at the relA Locus That Compensate the Detrimental Effects of Complete spoT Loss and Affect Glycogen Content in Escherichia coli
Source: PLoS One. 2014 Sep 4;9(9):e106938. doi: 10.1371/journal.pone.0106938 (PMC4154780; doi:10.1371/journal.pone.0106938)
Supplement: Table S1 — Oligonucleotides used for PCR constructions employed in the deletion of the E. coli spoT gene. spoT deletion was done following the method of Datsenko and Wanner [24]. Priming sequences for the Spc resistance gene are indicated in bold. (DOC) [file pone.0106938.s003.doc]

**Table S1:** Oligonucleotides used for PCR for the deletion of *spoT* gene following the method of Datsenko and Wanner (2000). Priming sequences for the Spc resistance gene are indicated in bold.

| Oligo | Locus | Orientation | Sequence |
| --- | --- | --- | --- |
| SPOT1 | +3bp of *spoT* ORF | sense | 5’-Tgctgaaggtcgtcgttaatcacaaagcgggtcgccct tg**acgaacCcagtggacataag**-3’ |
| SPOT2 | +2089 bp of *spoT* ORF | antisense | 5’-gtgttgggttcataaaacattaatttcggtttcgggtg ac**atgCat gatatatctcccaa**-3’ |
