# Supplementary material for: Systematic Production of Inactivating and Non-Inactivating Suppressor Mutations at the relA Locus That Compensate the Detrimental Effects of Complete spoT Loss and Affect Glycogen Content in Escherichia coli
Source: PLoS One. 2014 Sep 4;9(9):e106938. doi: 10.1371/journal.pone.0106938 (PMC4154780; doi:10.1371/journal.pone.0106938)
Supplement: Table S2 — Oligonucleotide primers used for PCR amplification of the different E. coli loci analyzed in this study. For further details see Figure S2. (DOC) [file pone.0106938.s004.doc]

**Table S2:** Oligonucleotides used for PCR amplification of different *E. coli* genes. For further details see **Figure S2**.

| **Oligo** | **Sequence** | **Annealing position** | **Orientation** |
| --- | --- | --- | --- |
| O1 | 5’-tacgccgaatatgattatctg- 3’ | -495 to -475 bp of *spoT* ORF | sense |
| O2 | 5’-CGAAGCCATGGTGCGCATGC- 3’ | +173 to +192 bp after *spoT*ORF stop codon | antisense |
| O3 | 5’-Acaccggaagggcgcattgtc- 3’ | +1174 to +1994 bp of *spoT* ORF | sense |
| O4 | 5’-TTAATTTCGGTTTCGGGTGACT TTAATC- 3’ | +2082 to +2109 bp of *spoT* ORF | antisense |
| O5 | 5’-GTGCAACCTGAAGATCGCG- 3’ | -500 to -481 bp of *relA* ORF | sense |
| O6 | 5’-TGCCATCCACCAGGTCAATC - 3’ | +179 to +198 bp after *relA* ORF stop codon | antisense |
| O7 | 5’-TGAGGGGGGCAACAAGGGG- 3’ | -495 to -477 bp of *rpoS* ORF | sense |
| O8 | 5’-TAGCAGACGGTCTGAAAACC- 3’ | +185 to +166 bp after *rpoS* ORF stop codon | antisense |
| O9 | 5’-CGCGTCCAGACTGCTATATTG- 3’ | -500 to -480 bp of *dksA* ORF | sense |
| O10 | 5’-TCTATGCGTACCAGCCAGCG- 3’ | +181 to +200 bp after *dksA* ORF stop codon | antisense |
| O11 | 5’-agttgctgttatcaaagcag- 3’ | -488 to -469 bp of *rpoB* ORF | sense |
| O12 | 5´-taacttcaccgaaagaccatg- 3’ | +171 to +191 bp after *rpoB* ORF stop codon | antisense |
| O13 | 5´-GATGGCAACAATAATGCAGG - 3´ | -499 to -480 bp of *rsd* ORF | sense |
| O14 | 5´-ACGACGGATGAAGCAAGAGAC- 3’ | +176 to +196 bp after *rsd* ORF stop codon | antisense |
| O15 | 5´- GCTTGTCTCTTCTCATCAG- 3´ | -467 to -449 bp of *ssrS* | sense |
| O16 | 5´- AATACAGCGACCGTATGTGC- 3’ | +175 to +194 bp after *ssrS* | antisense |
|  |  |  |  |
| O17 | 5’- TTGTCAGCCTGAAAATTCCG -3’ | -500 to -481 bp of *glnB* ORF | sense |
| O18 | 5’- AACTGGAAGGTGCGTTCAGC -3’ | +177 to +196 bp of *glnB* | antisense |
|  |  |  |  |
| O19 | 5’- CAAGCAGCAATAGTCAGTG -3’ | -500 to -482 bp of *yejB* ORF stop codon | sense |
| O20 | 5’- AACGGGAAATACCAACTGC -3’ | +157 to +175 bp after *yejB* ORF stop  codon | antisense |
|  |  |  |  |
| O21 | 5’- ATTAGCAGAGATGCGTTCCTC -3’ | -483 to -463 of *rpoZ* ORF | sense |
| O22 | 5’-GACCGAACGCAGCGGTGGTAAC-3’ | +224 to +245 of *rpoZ* ORF (within Spec resistance cassette) | antisense |
|  |  |  |  |
| O23 | 5’- TGCCGACTTCCGGTCAGATC -3’ | -495 to -476 of *rpoC* ORF | sense |
| O24 | 5’- ACAGGAATAGTATCGAATCC -3’ | +171 to +190 of *rpoC* ORF | antisense |
